# No monkey business: why studying NK cells in non-human primates pays off

**DOI:** 10.3389/fimmu.2013.00032

**Published:** 2013-02-18

**Authors:** Henoch S. Hong, Premeela A. Rajakumar, James M. Billingsley, R. Keith Reeves, R. Paul Johnson

**Affiliations:** ^1^Division of Immunology, New England Primate Research Center, Harvard Medical SchoolSouthborough, MA, USA; ^2^Infectious Disease Unit, Ragon Institute of Massachusetts General Hospital, MIT and Harvard, Massachusetts General HospitalBoston, MA, USA

**Keywords:** non-human primate models, rhesus macaques, NK cell subpopulations, transcriptional profiling

## Abstract

Human NK (hNK) cells play a key role in mediating host immune responses against various infectious diseases. For practical reasons, the majority of the data on hNK cells has been generated using peripheral blood lymphocytes. In contrast, our knowledge of NK cells in human tissues is limited, and not much is known about developmental pathways of hNK cell subpopulations *in vivo*. Although research in mice has elucidated a number of fundamental features of NK cell biology, mouse, and hNK cells significantly differ in their subpopulations, functions, and receptor repertoires. Thus, there is a need for a model that is more closely related to humans and yet allows experimental manipulations. Non-human primate models offer numerous opportunities for the study of NK cells, including the study of the role of NK cells after solid organ and stem cell transplantation, as well as in acute viral infection. Macaque NK cells can be depleted *in vivo* or adoptively transferred in an autologous system. All of these studies are either difficult or unethical to carry out in humans. Here we highlight recent advances in rhesus NK cell research and their parallels in humans. Using high-throughput transcriptional profiling, we demonstrate that the human CD56^bright^ and CD56^dim^ NK cell subsets have phenotypically and functionally analogous counterparts in rhesus macaques. Thus, the use of non-human primate models offers the potential to substantially advance hNK cell research.

## Introduction—the need for a better model to understand human NK cells

NK cells are lymphocytes that have evolved to provide a first line of immune protection against viruses and malignancies before adaptive immune responses emerge (Lanier, 2008). Increasing evidence suggests that human NK (hNK) cell contributions to the host defense against viruses are pivotal. People with genetic NK cell deficiencies, though very rare, display severe primary or recurring infections by members of the herpesvirus family (Orange, 2006), suggesting that NK cells are crucial to host defense against viral infections. In addition, epidemiological data suggest a protective role for NK cells in hepatitis C virus infection (Khakoo et al., 2004) and HIV infection (Martin et al., 2002, 2007). Although these studies suggest that a better knowledge of hNK cells has the potential to be translated into novel, therapeutic approaches, research has been hindered by the limitations of the human “model.” Most of our knowledge on hNK cells has been derived from studies performed on peripheral blood lymphocytes due to ease of accessibility. However, NK cells in blood only represent one specialized subset of the total hNK cell compartment. Furthermore, manipulations of the human immune system are either highly challenging and/or unethical to carry out and are thus largely limited to vaccination.

The study of murine NK (mNK) cells has clearly elucidated a number of fundamental principles, some of which seem to universally apply to all NK cells, such as the activation of NK cells by absent or altered MHC class I molecule expression, also known as the “missing self hypothesis” (Karre et al., 1986; Ljunggren and Karre, 1990). Recent pioneering work suggests that mNK cells can exhibit adaptive immune features, a paradigm-altering concept, which has not yet been observed in hNK cells (Sun et al., 2009; Paust et al., 2010). Whereas mice continue to serve as a powerful tool to study basic immunological questions, many research advances made in murine models have not been translated into medical practice (Davis, 2008). In part, this discrepancy may be explained by the substantial differences between mouse and human immunology, which is not surprising given the evolutionary distance of 65–75 million years that separate human and mice development (Mestas and Hughes, 2004), as well as the challenges of modeling human diseases in murine models.

There are a number of important differences between hNK and mNK cells. In mice, cognate MHC class I molecules are recognized by Ly49 protein family members, which contain C-type lectin domains (Carlyle et al., 2008). In contrast, interactions of hNK cells with classical MHC class I molecules rely primarily on killer cell immunoglobulin-like receptors (KIRs). Although Ly49 proteins and KIRs are functional homologs, they are different in almost every other aspect, including structural properties, different binding sites of the MHC class I molecules, and genetic differences (Natarajan et al., 2002; Pascal et al., 2006). Furthermore, despite a remarkably high degree of conservation of NKG2D in humans compared to mice, phylogenetic analyses suggest that the ligands diversified independently from each other (Raulet, 2003; Eagle and Trowsdale, 2007).

Importantly, there are also differences between hNK and mNK cell subpopulations. In humans, two phenotypically and functionally distinct peripheral blood NK cell subsets have been described based on the expression of CD56 and CD16 (Caligiuri, 2008). The predominant population of CD16^+^CD56^dim^ NK cells is known for its cytolytic activity and limited cytokine production (Lanier et al., 1986). In contrast, the less frequent CD56^bright^ NK cell subset does not express CD16 and is traditionally considered to possess “regulatory” functions (Cooper et al., 2001). Although the use of CD27 and Mac-1 (CD11b) allows mature mNK cells to be divided into distinct subsets with some parallels to hNK cell subsets (Hayakawa and Smyth, 2006), there remain significant differences between mNK and hNK cell subpopulations, such as divergent expression of CD62L, CCR7, CX3CR1, and other phenotypic markers (Hayakawa et al., 2006). Also, to our knowledge it is not known whether CD27^low^ NK cells mediate antibody-dependent cytotoxic activity as CD56^dim^ NK cells do in humans.

Here, we argue that non-human primate models, such as rhesus macaques, represent a powerful animal model with significant potential for the study of hNK cells for two important reasons. First, rmNK cells exhibit far greater similarities to hNK cells than mNK cells. Second, as an animal model, non-human primates allow ready access to tissues and experimental manipulations that are highly challenging or unethical to carry out in humans.

## Defining NK cell subpopulations in rhesus macaques

Unlike hNK cells, virtually all rmNK cells in peripheral blood express CD8α and most of them do not express CD56 (Carter et al., 1999). In addition, not all peripheral rmNK cells express NKp46 (Reeves et al., 2010) but virtually all of them express NKG2A, which renders this receptor a highly reliable marker for the definition of peripheral rmNK cells (Mavilio et al., 2005a; Webster and Johnson, 2005). Thus, after gating on CD3^−^CD8α^+^NKG2A^+^ cells, analysis of CD56 and CD16 allows the definition of 3 distinct NK cell subsets in rhesus macaques: CD56^+^, CD56^−^CD16^−^ double-negative (DN), and CD16^+^ NK cells (Webster and Johnson, 2005; Reeves et al., 2010). Rigorous NK cell phenotyping revealed that the CD56^+^CD16^−^ population resembles CD56^bright^ hNK cells, especially with regard to expression of lymph node homing markers, such as CCR7 and CD62L, in addition to lower expression of granzyme B and perforin (Webster and Johnson, 2005; Reeves et al., 2010). Conversely, the CD16^+^ rmNK cell population corresponds well to the CD56^dim^ hNK cell subset, as evidenced by the increased expression of granzyme B and perforin and absence of CCR7 and CD62L. A relatively infrequent CD161^+^ DN hNK cell population has been defined (Bennett et al., 1996) but it remains unclear whether this population corresponds to the DN rmNK cell subset.

## Transcriptional analysis reveals a high degree of homology between rhesus and hNK cells

A previous transcriptome analysis study revealed a number of differentially expressed genes in CD56^bright^ and CD56^dim^ hNK cells (Hanna et al., 2004). We therefore tested the notion that CD56^+^ and CD16^+^ rmNK cells correspond to CD56^bright^ and CD56^dim^ hNK cells by performing an extensive transcriptional analysis of rmNK cell subpopulations using a high-throughput microfluidics PCR platform (Fluidigm BioMark) on highly purified CD56^+^, CD16^+^, and DN rmNK cells. We either utilized rhesus-specific ABI TaqMan assays, when available, (Life Technologies), or custom-designed primers and probes based on rhesus mRNA sequences. A complete list of real-time PCR assays employed in our study is available upon request. All assays were subjected to a rigorous selection process to ensure that targeted rhesus macaque genes were orthologous to human genes.

**Figure 1:**
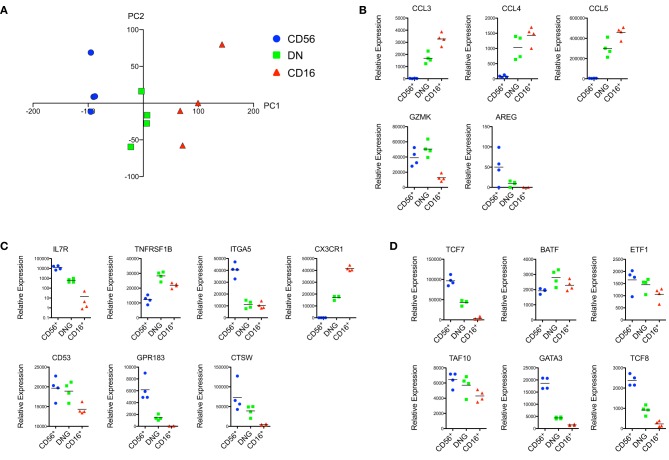
**Transcriptional data suggest a high degree of homology between hNK and rmNK cell subsets. (A)** Principal component analysis (PCA) representation of the CD56^+^, DN, and CD16^+^ rmNK cell subsets. PC1 (horizontal) and PC2 (vertical) axes are shown. **(B–D)** Relative mRNA expression of genes encoding for effector proteins **(B)**, cell surface receptors and proteins **(C)** and proteins associated with transcriptional control **(D)** are shown.

Principal component analysis (PCA) revealed a segregation of NK cells into groups corresponding to the CD56^+^, CD16^+^, and DN rmNK cell subsets (Figure 1A). Similar results were observed when we subjected the data to unsupervised hierarchical clustering (data not shown). We analyzed the relative expression of a number of effector proteins and found low expression of the β-chemokines CCL3, CCL4, and CCL5 in CD56^+^ cells and high expression of these genes in CD16^+^ cells (Figure 1B). Conversely, transcripts for granzyme K (GZMK) and amphiregulin (AREG) were more abundantly found in CD56^+^ cells. We found high mRNA expression of the IL-7R in CD56^+^ cells but negligible expression in CD16^+^ cells (Figure 1C) as expected based on human microarray and flow cytometric data (Hanna et al., 2004; Vosshenrich et al., 2006). In addition, we were able to identify predicted expression patterns for the tumor necrosis factor receptor superfamily member 1B (TNFRSF1B), integrin α5 (ITGA5), CX3CR1, CD53, G protein-coupled receptor 183 (GPR183), and cathepsin W (CTSW) (Figure 1C). Finally, the transcription factors TCF7, ETF1, GATA3, and TCF8 were highly expressed in CD56^+^ compared to CD16^+^ rmNK cells, whereas the reverse trend was observed for BATF (Figure 1D).

Notably, the DN NK cell subset represented an intermediate population between CD56^+^ and CD16^+^ NK cells, both in the PCA analysis as well as for most of the gene expression data presented in Figure 1. This is of particular interest since increasing evidence suggests that CD56^bright^ NK cells represent a less mature developmental stage of NK differentiation, whereas CD56^dim^ cells exhibit a more differentiated effector profile (Romagnani et al., 2007; Yu et al., 2010; Beziat et al., 2011). We observed similar patterns in rmNK cells, as evidenced by expression of IL-7R and cKIT (data not shown), and TCF7 in CD56^+^ cells, a pattern consistent with a more primitive stage of differentiation. Conversely, the expression of effector proteins such as CCL3, CCL4, and CCL5 was predominantly found in CD16^+^ cells. In this context, DN NK cells are likely to represent an intermediate stage of NK cell differentiation between CD56^+^ and CD16^+^ cells. A number of potential candidates have been suggested as intermediate NK cell populations in humans, including CD16^+^CD56^bright^ cells (Beziat et al., 2011), CCR7^−^CD56^bright^ cells (Hong et al., 2012), CD94^bright^CD56^dim^ cells (Yu et al., 2010), and CD62L^+^CD56^dim^ cells (Juelke et al., 2010). These studies reflect the dynamic nature of NK cell differentiation and emphasize the need for additional experimental evidence to rigorously establish the ontogeny of these subsets.

**Figure 2:**
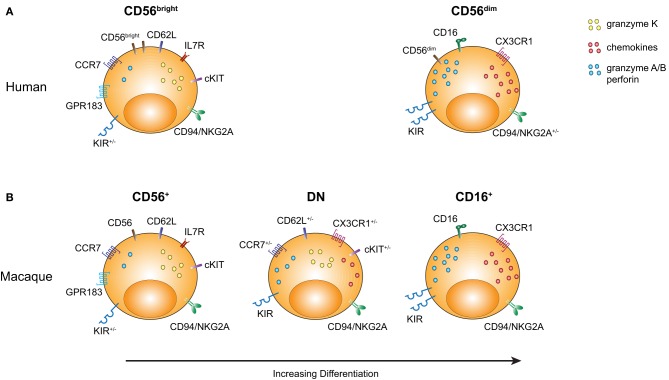
**Comparison of rmNK and hNK cell subpopulations.** Markers characterizing the distinct NK cell subpopulations are illustrated with an emphasis on molecules either indicating a more primitive stage of hematopoietic differentiation or an effector cell profile. **(A)** Human CD56^bright^ and CD56^dim^ NK cells are shown. **(B)** Rhesus CD56^+^, DN, and CD16^+^ NK cell subsets are shown.

In summary, based on the available phenotypic and transcriptional profiling data, we suggest that the rmNK CD56^+^ and CD16^+^ populations bear a striking homology to their hNK CD56^bright^ and CD56^dim^ counterparts (Figure 2). Moreover, the rhesus DN NK cell population displays a distinctive pattern of gene expression that strongly suggests it represents an intermediate stage of differentiation between CD56^+^ and CD16^+^ NK cells.

## Human and non-human primate NK cells in HIV and SIV infection

The discovery of simian immunodeficiency virus (SIV) in rhesus macaques with an acquired and transmissible immunodeficiency (Daniel et al., 1985) generated considerable interest in non-human primate species as disease models for HIV. Non-human primates have since served as an invaluable resource to study the pathogenesis of lentiviral infections and to test vaccine and treatment strategies against HIV (Van Rompay, 2012).

Multiple phenotypic and functional changes within the NK cell compartment have been documented in HIV infection (Fauci et al., 2005; Iannello et al., 2008) and many similar alterations have been identified in SIV infection (Bostik et al., 2010; Reeves et al., 2010). One of the hallmarks of HIV and pathogenic SIV infection is systemic immune activation of the host (Douek et al., 2009). This is reflected by the activation states of virtually all immune cells, including NK cells, as shown by increased expression of activation markers and functional hyperactivity as detected by increased frequencies of NK cells producing IFN-γ, TNF-α and enhanced degranulation and killing activity (Giavedoni et al., 2000; Alter et al., 2004; Fogli et al., 2004). Acute HIV and SIV infection were found to be associated with an initial expansion followed by a contraction of NK cells (Giavedoni et al., 2000; Alter et al., 2007). Although NK cells were generally shown to proliferate and die more rapidly than T cells in humans (Lutz et al., 2011), their turnover rates are further enhanced in chronic HIV infection (Kottilil et al., 2007). Increased NK cell turnover was also detected in SIV-infected rhesus macaques in a study in which NK cells were defined as CD3^−^CD8^+^CD16^+^ cells (De Boer et al., 2003), although this gating strategy excludes the CD56^+^ and DN rmNK cell subpopulations, and may have limited the authors' ability to accurately assess the effects of SIV infection on the full repertoire of rmNK cells.

Although NK cells from chronically HIV- or SIV-infected donors display a hyper-activated state, their capacity to respond to PMA and ionomycin is diminished (Azzoni et al., 2002; Labonte et al., 2006). Furthermore, studies have demonstrated a dramatic skewing of NK cell subpopulations in peripheral blood during SIV and HIV infection. HIV infection was associated with a decrease of CD56^dim^ NK cells and an increase of the CD56^−^CD16^+^ NK cell subset (Alter et al., 2005; Mavilio et al., 2005b; Hong et al., 2010a,b), whereas SIV-infected macaques displayed an expansion of DN and CD16^+^ NK cells (Reeves et al., 2010). Interestingly, both HIV and SIV infection induced a loss of CCR7-expressing CD56^bright^ and CD56^+^ cells, respectively, without affecting the frequencies of cells expressing CD62L (Reeves et al., 2010; Hong et al., 2012). This perturbation was furthermore accompanied by an increased expression of granzyme B and perforin and elevated degranulation in response to MHC class I-devoid tumor cells (Mantegani et al., 2009). Importantly, there seems to be an inverse relationship between the severity of immune activation and/or viral load and the impact of viral infection on NK cells, as evidenced in natural host SIV infection in sooty mangabeys (Pereira et al., 2008) or non-viremic HIV-patients (Vieillard et al., 2010). Taken together, these studies provide solid evidence for SIV-induced perturbations in rmNK cells similar to what has been described in chronic HIV infection.

## Non-human primate studies shed light on NK cells in tissues

Whereas most hNK studies have focused on NK cell subsets from peripheral blood, much less is known about NK cells in tissues. The complexity and heterogeneity of hNK cells residing in various organs, such as uterus, brain, spleen, liver, pancreas, and skin, as well as various mucosal tissues, including the gut, is an area of research that is garnering increasing interest (Shi et al., 2011). For obvious reasons, research is hindered by the significant limitations in obtaining human tissue samples. Here, we highlight one example of how studies on tissue-residing NK cells in non-human primates may lead to a better understanding of human diseases.

A novel IL-22- and IL-17-producing NKp44^+^ hNK cell subset was recently discovered in human and mouse mucosal-associated lymphoid tissues (Cella et al., 2009; Crellin et al., 2010). Unlike conventional NK cells, these lymphocytes do not display cytolytic activity but instead seem to be crucial for maintaining the integrity of epithelial tissues. Since HIV and SIV are predominantly transmitted and replicate in gut-associated lymphoid tissues (GALT), we and others sought to explore the role of IL-22-producing NK cells in SIV infection. SIV infection was associated with a substantial loss of IL-17- and IL-22-producing lymphocytes, including NKp44^+^ NK cells (Reeves et al., 2011; Klatt et al., 2012; Xu et al., 2012). Remarkably, the remaining NKp44^+^ NK cells displayed an altered functional profile with increasing resemblance to conventional NK cells. These alterations were linked to gut inflammation and the up-regulation of indoleamine 2,3-dioxygenase 1 (Reeves et al., 2011). Given the importance of IL-17 and IL-22 for the maintenance of gut integrity and enterocyte homeostasis, these studies suggest a mechanistic explanation of how HIV and SIV infection damage the epithelium and subsequently drive disease progression.

A recent study described an expansion of intraepithelial and lamina propria NKp46^+^ NK cell subsets in treated HIV-patients with incomplete or suboptimal CD4^+^ T cell recovery (Sips et al., 2012). This finding suggests that gut mucosa-residing NKp46^+^ NK cells could play a compensatory role in patients with ongoing compromised immunity. However, to our knowledge, the impact of HIV infection on IL-22-producing NKp44^+^ hNK cells has not yet been addressed.

## Manipulations of the non-human primate immune system

In addition to improved access to tissue samples, a number of experimental procedures that have the potential to significantly advance our understanding of NK cells can be carried out in non-human primates. For instance, the *in vivo* effects of cytokines on lymphocyte homeostasis and disease can be addressed in rhesus macaques (Kuramoto et al., 2004; Waldmann et al., 2011). Administration of IL-15 resulted in an almost 3-fold increase of circulating NK cells in addition to increasing the number of effector memory CD8^+^ T cells (Mueller et al., 2005). Rhesus and cynomolgus macaque models have been a critical tool for the study of immune responses in the setting of transplantation (Kean et al., 2012). NK cells can be involved in both graft rejection and induction of tolerance (Kroemer et al., 2008). Efforts to therapeutically modulate NK cells to facilitate tolerance against allogeneic tissue grafts are likely to require further studies in animal models and, in particular, in non-human primates.

To evaluate the antiviral contributions of NK cells in control of SIV infection, mouse monoclonal antibodies against CD16 have been administered to rhesus macaques (Choi et al., 2008). There are several caveats to this approach, one of them being that the majority of tissue NK cells, in contrast to NK cells in peripheral blood, do not express CD16 (Reeves et al., 2010). Nonetheless, this study and other lymphocyte depletion studies (Schmitz et al., 1999), provide ample evidence that the depletion of selected lymphocyte populations, including NK cells, is possible and can thus be utilized to study their role in an *in vivo* setting.

Furthermore, non-human primates could also be used in autologous transfer studies using fluorescently labeled cells and *ex vivo* expanded cells. This approach could generate novel insights into NK cell turnover, differentiation, migratory behavior, *in vivo* killing of target cells, and other areas. Moreover, induced pluripotent stem cells have been used to generate hNK cells with antiviral activity against HIV (Knorr and Kaufman, 2010). NK cells derived from induced rhesus pluripotent stem cells (Liu et al., 2008) could be used to monitor *in vivo* NK cell development and differentiation. These and many other experimental manipulations of the immune system in non-human primates open a number of new avenues to address basic immunological questions and highlight the potential of these animal models.

## Conclusion

Here we present two arguments as to why NK cell research in non-human primate models has the potential to yield significant insights into hNK biology. First, as a consequence of the phylogenetic relatedness of humans and non-human primates, there are many shared properties between hNK and non-human primate NK cells. Second, as an animal model, non-human primates offer access to tissues and allow a variety of manipulations, which the “human model” cannot offer due to ethical constraints. The work in non-human primates is challenging, as animals and their housing are relatively cost-intensive and require dedicated facilities and staff. Nonetheless, we believe that their significance as a disease model and the potential clinical applicability of immunological findings derived from these animals continue to make research in non-human primates highly rewarding.

Non-human primate models not only bear relevance for humans as outstanding disease models but also as a valuable resource to address basic immunological questions. The exciting answers to these questions and the lessons non-human primates can teach us certainly deserve a greater consideration by the scientific community.

### Conflict of interest statement

The authors declare that the research was conducted in the absence of any commercial or financial relationships that could be construed as a potential conflict of interest.
